# Supplementary material for: Novel application of the ferroptosis-related genes risk model associated with disulfidptosis in hepatocellular carcinoma prognosis and immune infiltration
Source: PeerJ. 2024 Feb 2;12:e16819. doi: 10.7717/peerj.16819 (PMC10840499; doi:10.7717/peerj.16819)
Supplement: Supplemental Information 1 [file peerj-12-16819-s001.docx]

supplementary materials

| 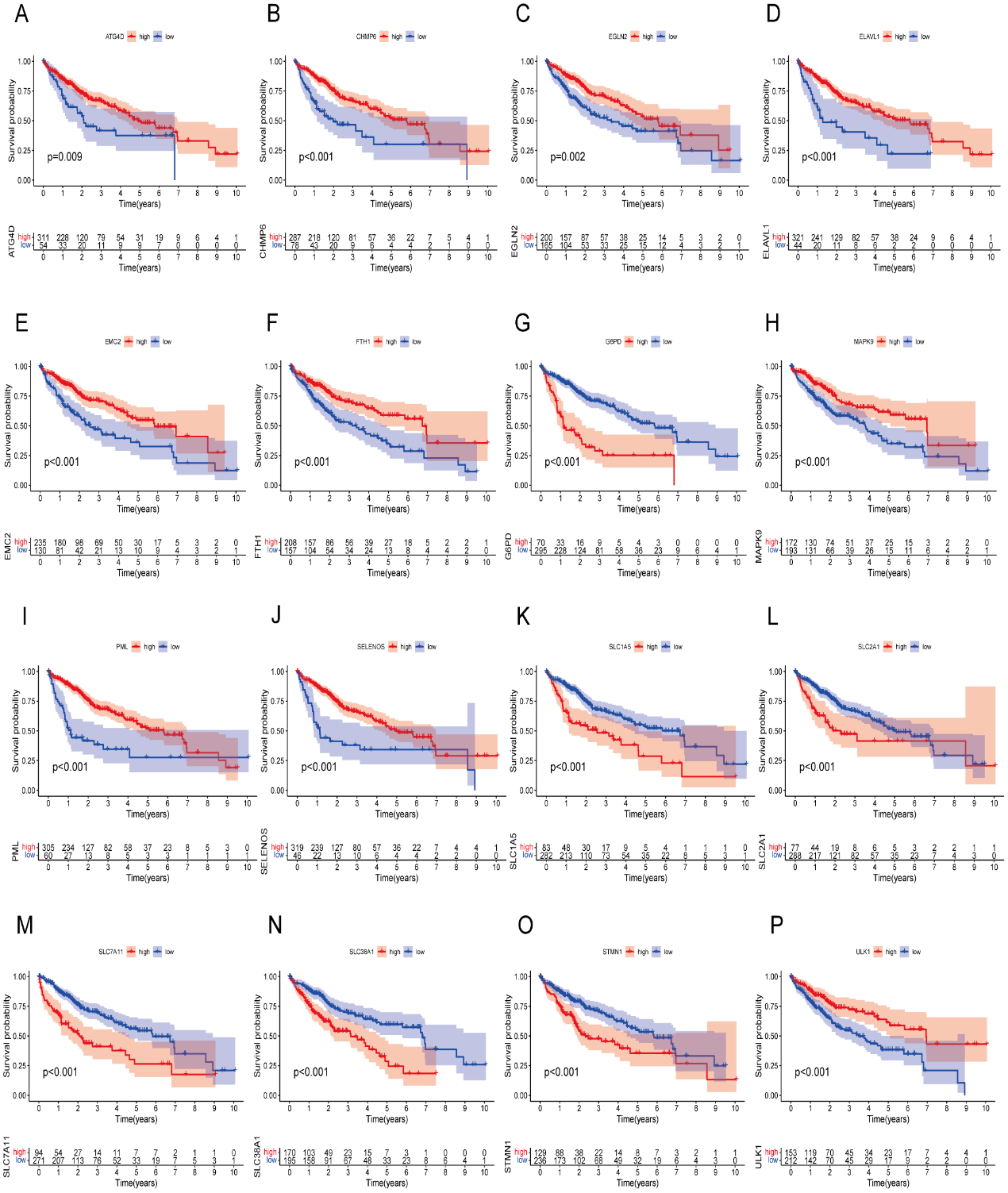 |
| --- |
| Figure S1 Univariate Cox regression analysis of 16 DRG-FRGs in the TCGA cohort. (A-P) 16 DRG-FRGs were all independently associated with OS. DRG-FRGs, ferroptosis-related genes associated with disulfidptosis-related genes; OS, overall survival. |

| 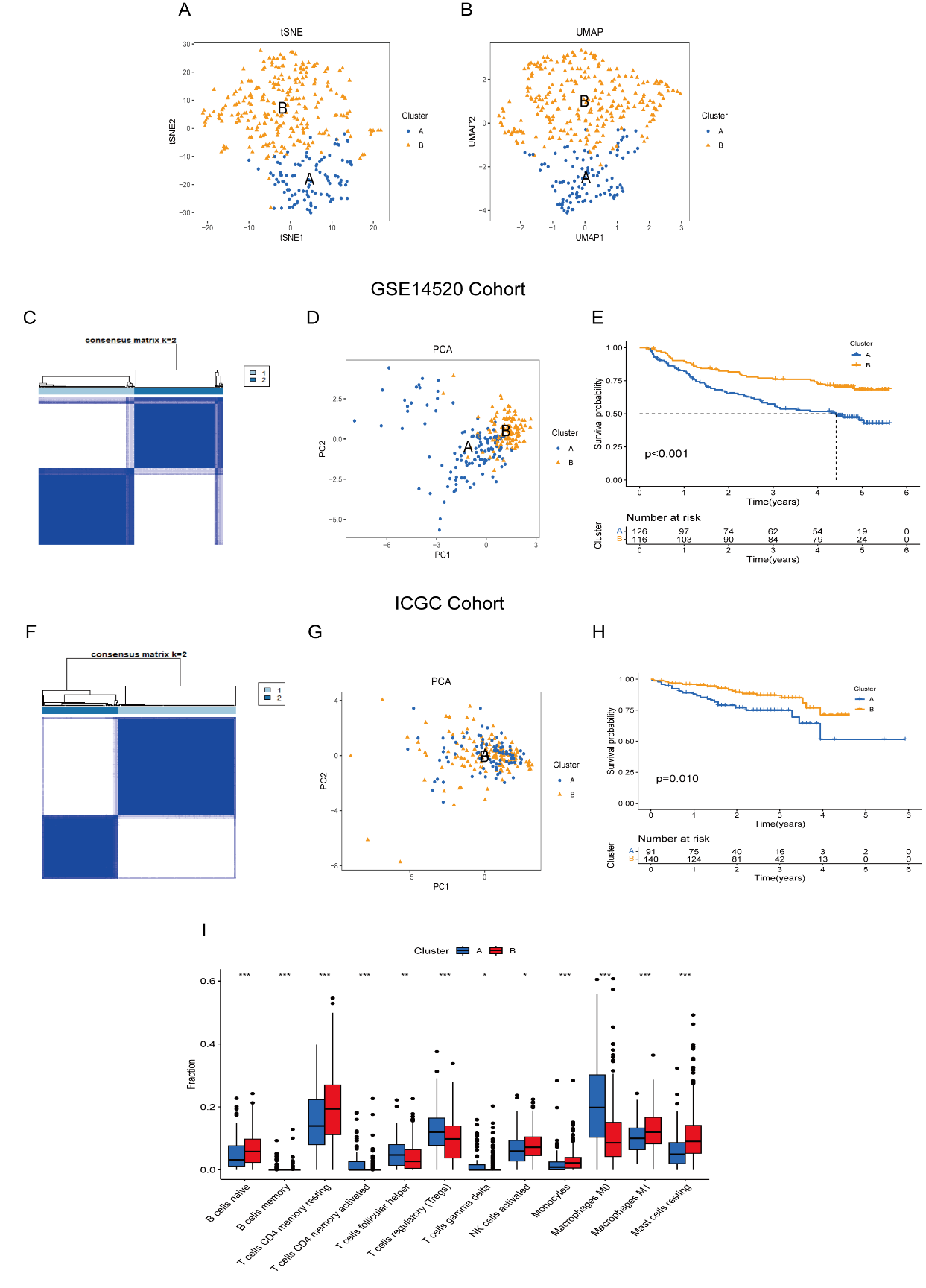 |
| --- |
| Figure S2 Clustering analysis and immune infiltration analysis in the TCGA and external cohorts. (A, B) t-SNE, UMAP algorithm to identify the differentiation of two clusters in the TCGA cohort. (C, F) Clustering of 16 DRG-FRGs and optimal consensus matrices for k = 2 in the GSE14520 and ICGC cohorts. (D, G) PCA analysis of the two clusters in the GSE14520 and ICGC cohorts. (E, H) Survival analysis of the two clusters in the GSE14520 and ICGC cohorts. (I) Comparison of immune cell infiltration in two clusters in the TCGA cohort. PCA, principal component analysis; t-SNE, t-distributed stochastic neighbor embedding; UMAP, uniform manifold approximation and projection. DRG-FRGs, ferroptosis-related genes associated with disulfidptosis-related genes. ∗ *P* < 0.05; ∗∗ *P* < 0.01; ∗∗∗ *P* < 0.001. |

| 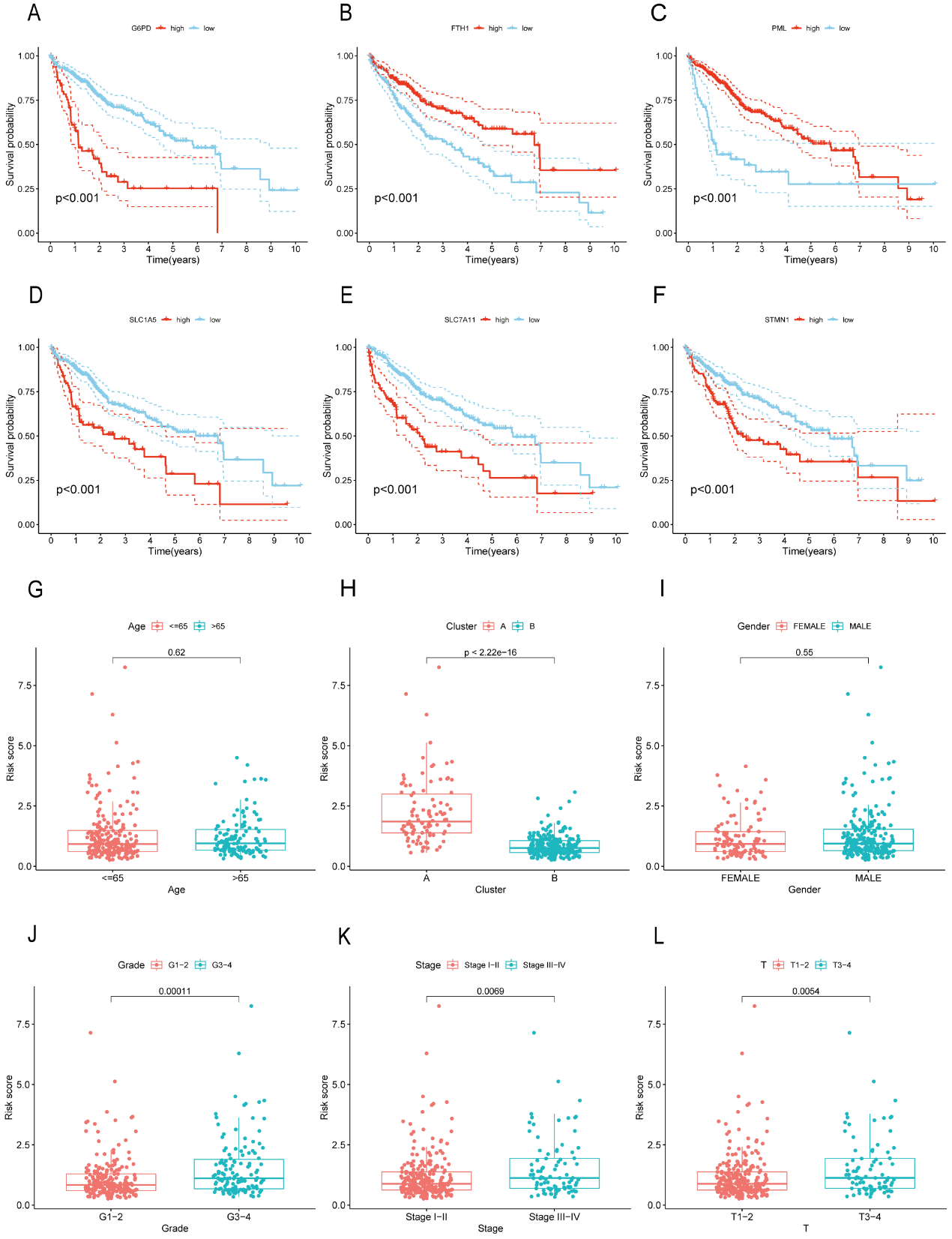 |
| --- |
| Figure S3 Survival analysis of 6 signature genes and distribution of risk scores in HCC with different clinical features in the TCGA cohort. (A-F) Kaplan-Meier curves for 6 signature genes in the TCGA cohort. (G-L) The distribution of risk scores in HCC with different ages, clusters, genders, grades, and stages in the TCGA cohort. |

| 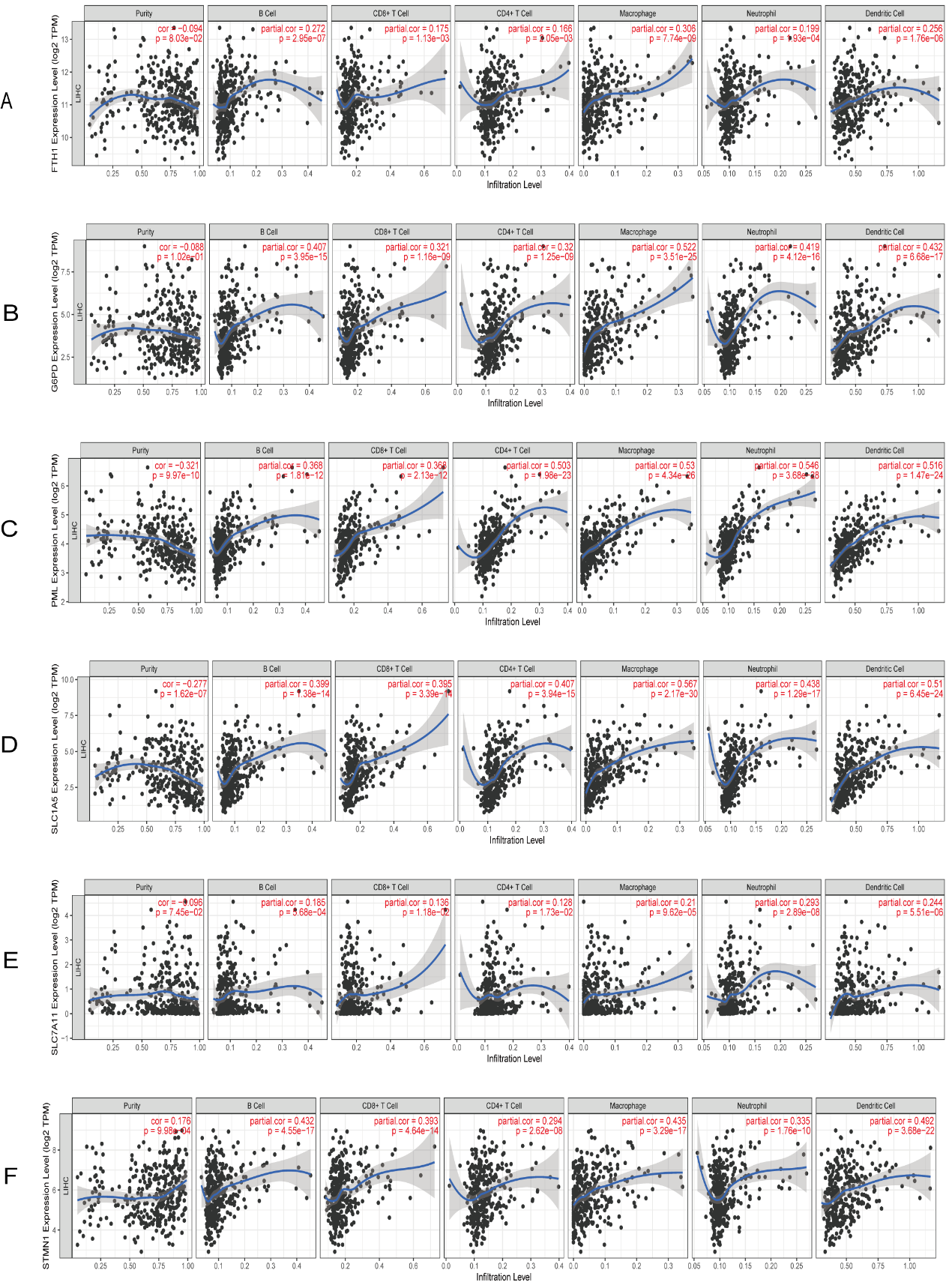 |
| --- |
| Figure S4 The correlation between 6 signature genes and immune cell infiltration in the TCGA cohort. (A) FTH1. (B) G6PD. (C) PML. (D) SLC1A5. (E) SLC7A11. (F) STMN1. |

| 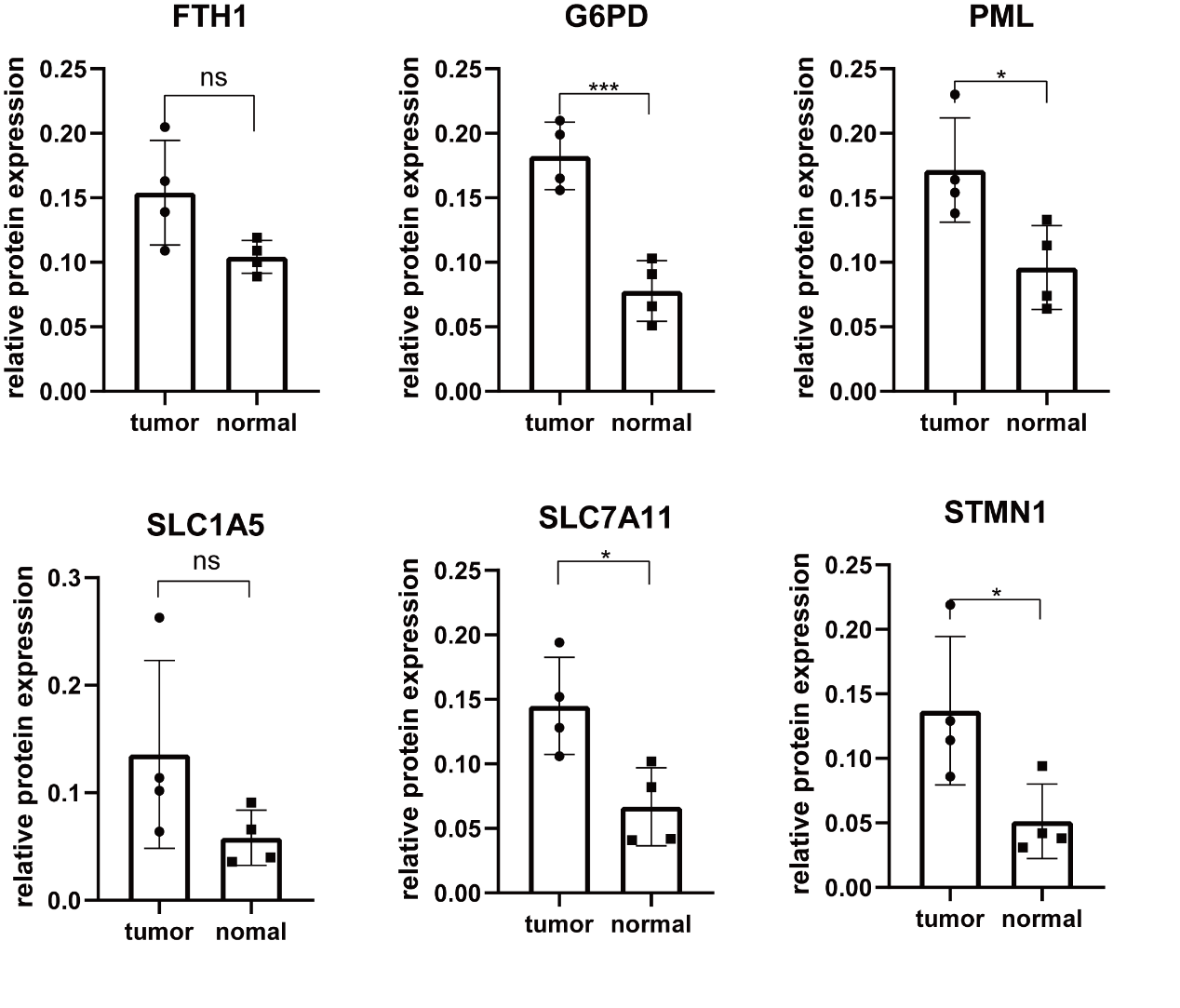 |
| --- |
| Figure S5 The expression difference of the corresponding proteins of 6 signature genes in 24 pairs of HCC tissues and paired adjacent tissues. (A) FTH1. (B) SLC1A5. (C) PML. (D) G6PD. (E) SLC7A11. (F) STMN1. ns, no significant difference; ∗ *P* < 0.05; ∗∗∗ *P* < 0.001. |
